# iTRAQ-Based Proteomic Analysis of Ginsenoside F_2_ on Human Gastric Carcinoma Cells SGC7901

**DOI:** 10.1155/2016/2635483

**Published:** 2016-10-18

**Authors:** Qian Mao, Pin-Hu Zhang, Jie Yang, Jin-Di Xu, Ming Kong, Hong Shen, He Zhu, Min Bai, Li Zhou, Guang-Fu Li, Qiang Wang, Song-Lin Li

**Affiliations:** ^1^Department of Pharmaceutical Analysis & Metabolomics, Affiliated Hospital of Integrated Traditional Chinese and Western Medicine, Nanjing University of Chinese Medicine, Nanjing 210028, China; ^2^Jiangsu Center for New Drug Screening & National New Drug Screening Laboratory, China Pharmaceutical University, Nanjing 210009, China; ^3^Department of Chinese Medicines Analysis, China Pharmaceutical University, Nanjing 210009, China; ^4^Department of Surgery, The Medical University of South Carolina, Charleston, SC 29466, USA

## Abstract

Ginsenoside F_2_ (F_2_), a protopanaxdiol type of saponin, was reported to inhibit human gastric cancer cells SGC7901. To better understand the molecular mechanisms of F_2_, an iTRAQ-based proteomics approach was applied to define protein expression profiles in SGC7901 cells in response to lower dose (20 *μ*M) and shorter duration (12 hour) of F_2_ treatment, compared with previous study. 205 proteins were screened in terms of the change in their expression level which met our predefined criteria. Further bioinformatics and experiments demonstrated that F_2_ treatment downregulated PRR5 and RPS15 and upregulated RPL26, which are implicated in ribosomal protein-p53 signaling pathway. F_2_ also inhibited CISD2, Bcl-xl, and NLRX1, which are associated with autophagic pathway. Furthermore, it was demonstrated that F_2_ treatment increased Atg5, Atg7, Atg10, and PUMA, the critical downstream effectors of ribosomal protein-p53 signaling pathway, and Beclin-1, UVRAG, and AMBRA-1, the important molecules in Bcl-xl/Beclin-1 pathway. The 6 differentially abundant proteins, PRR5, CISD2, Bcl-xl, NLRX1, RPS15, and RPL26, were confirmed by western blot. Taken together, ribosomal protein-p53 signaling pathway and Bcl-xl/Beclin-1 pathway might be the most significantly regulated biological process by F_2_ treatment in SGC7901 cells, which provided valuable insights into the deep understanding of the molecular mechanisms of F_2_ for gastric cancer treatment.

## 1. Introduction

Gastric cancer is the fifth most common cancer and the third leading cause of cancer-related death worldwide. Annually it results in approximately 700,000 deaths [1]. Currently, chemotherapy has proved to decrease the rate of recurrence and improve overall survival; however, the drug resistance and serious toxic side effects largely reduce therapeutic efficacy and quality of life in patients [2, 3]. In recent years, compounds of natural products have caught wide attention due to their promising anticancer effects and minimal side effects [4–7]. Therefore, it is very necessary to develop new optimal anticancer agent from natural resource [3].

**Figure 1:**
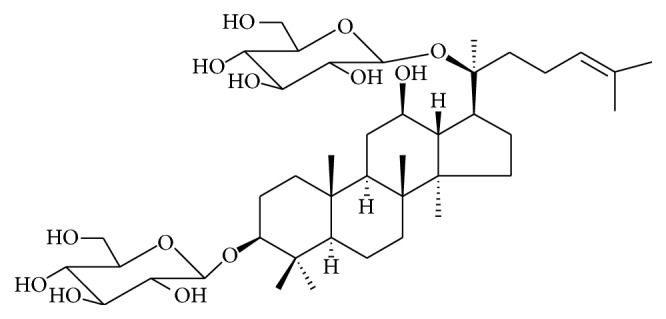
Structure of ginsenoside F_2_.

Ginsenosides, the major bioactive constituents in ginseng, have been demonstrated to exert potential anticancer ability [4, 5]. Exploration of ginsenoside as a new anticarcinogenic agent is of much interest [4–7]. Structural-function studies showed that the increased antitumor effect is implicated with the decrease of its sugar number [5]. Sugar moiety at C-6 significantly reduces the anticancer activities of ginsenosides. Ginsenoside F_2_ (see structure in Figure 1), a protopanaxdiol type ginsenoside with one sugar molecular at C-3 and one sugar molecule at C-20, has been shown to be potent in inhibiting tumorigenesis in several different cancers including gastric tumor and glioblastoma multiforme [6, 7]. Recently, our* in vitro* and* in vivo* studies demonstrated that ginsenoside F_2_ possesses anticancer effects in human gastric carcinoma cells SGC7901 [6]. However, the involved exact mechanisms of ginsenoside F_2_ on SGC7901 cancer cells at proteome level have not been systemically investigated.

Advancements in the field of proteomics have made it possible to accurately monitor and quantitatively detect the changes of protein expression in response to drug treatment. The achieved data provide valuable insights into the molecular mechanisms of disease and help to identify therapeutic targets [8]. Isobaric tag for relative and absolute quantification (iTRAQ) is a robust mass spectrometry technique that allows quantitative comparison of protein abundance by measuring peak intensities of reporter ions released from iTRAQ-tagged peptides by fragmentation. iTRAQ with multiplexing capability up to eight distinct samples in a single experiment and relatively higher sensitivity has gained significant interest in the field of quantitative proteomics. In the present study, SGC7901 cells treated by lower dose and a shorter duration than that in previous report were analyzed by iTRAQ-based proteomics integrated with bioinformatics using Gene Ontology (GO), Kyoto Encyclopedia of Genes and Genomes (KEGG), and Cluster of Orthologous Groups (COG) of proteins database. And network analysis was applied to identify critical molecules which are involved in anticancer mechanisms of ginsenoside F_2_ in gastric SGC7901 cells. General molecular biological techniques such as western blot were utilized for validation.

## 2. Materials and Methods

### 2.1. Reagents and Antibodies

Ginsenoside F_2_ was isolated previously from leaves of* Panax ginseng* by a series of chromatographic procedures [9]. Ginsenoside F_2_ has a molecular mass of 784 Da and was isolated with 98% purity. Primary antibodies of PRR5, CISD2, Bcl-2L, NLRX1, RPS15, RPL26, p53, PUMA, Beclin-1, UVRAG, AMBRA-1, mTOR, LC3-II, LC3-I, and *β*-actin together with all secondary antibodies were purchased from Cell Signaling Technology (Danvers, MA, USA). The Atg5, Atg7, and Atg10 antibodies were obtained from Santa Cruz Biotechnology (Santa Cruz, CA, USA).

### 2.2. Cell Culture and Treatment

SGC7901 cells were purchased from American Type Culture Collection and maintained in Dulbecco's modified Eagle's medium (Hyclone) supplemented with 10% fetal bovine serum (FBS), 100 *μ*g/mL streptomycin, and 100 *μ*g/mL penicillin and grown at 37°C in 5% carbon dioxide.

### 2.3. Protein Preparation

In one of our recent reports [6], we have shown that the IC_50_ of ginsenoside F_2_ is in <50 *μ*M in 24 hours. In order to characterize ginsenoside F_2_-related mechanism it is imperative to use samples that are at the early stages of ginsenoside F_2_ treatment. So, a lower dose than the IC_50_ (20 *μ*M) and a shorter duration (12 hours in the study) were chosen in the study. The treated (20 *μ*M) and untreated SGC7901 cells were suspended in the lysis buffer and sonicated in ice. The proteins were reduced with 10 *μ*M DTT (final concentration) at 56°C for 1 h and then alkylated by 55 mM iodoacetamide (IAM) (final concentration) in the darkroom for 1 h. The reduced and alkylated protein mixtures were precipitated by adding 4x volume of chilled acetone at −20°C overnight. After centrifugation at 4°C, 30 000 ×g, the pellet was dissolved in 0.5 M triethylammonium bicarbonate (TEAB) (Applied Biosystems, Milan, Italy) and sonicated in ice. After centrifuging at 30000 ×g at 4°C, the supernatants were collected, and the total protein concentration was determined using a Bradford protein assay kit (BioRad, Hercules, CA, USA). The proteins in the supernatant were kept at −80°C for further analysis.

### 2.4. iTRAQ Labeling and SCX Fractionation

Total protein (100 *μ*g) was taken out of each sample solution and then the protein was digested with Trypsin Gold (Promega, Madison, WI, USA) with the ratio of protein : trypsin = 30 : 1 at 37°C for 16 hours. iTRAQ labeling was performed according to the iTRAQ Reagents-8plex labeling manual (AB SCIEX, Madrid, Spain). Briefly, one unit of iTRAQ reagent was thawed and reconstituted in 24 *μ*L isopropanol. iTRAQ labels 113 were used to label control sample separately, and 115 and 117 were used to label twice F_2_-treated samples for duplicated experiment. The peptides were labeled with the isobaric tags, incubated at room temperature for 2 h. The labeled peptide mixtures were then pooled and dried by vacuum centrifugation.

The mixed peptides were fractionated by strong cation exchange (SCX) chromatography on a LC-20AB HPLC Pump system (Shimadzu, Kyoto, Japan). The iTRAQ labeled peptide mixtures were reconstituted with 4 mL buffer A (25 mM NaH_2_PO_4_ in 25% acetonitrile, pH 2.7) and loaded onto a 4.6 × 250 mm Ul tremex SCX column containing 5 *μ*m particles (Phenomenex). The peptides were eluted at a flow rate of 1 mL/min with a gradient of buffer A for 10 min, 5–60% buffer B (25 mM NaH_2_PO_4_, 1 M KCl in 25% acetonitrile, pH 2.7) for 27 min, and 60–100% buffer B for 1 min. The system was then maintained at 100% buffer B for 1 min before equilibrating with buffer A for 10 min prior to the next injection. Elution was monitored by measuring the absorbance at 214 nm, and fractions were collected at 1-minute intervals. The eluted peptides were pooled into 20 fractions, desalted with a Strata X C18 column (Phenomenex), and vacuum-dried. The cleaned fractions were then lyophilized again and stored at −20°C until analyzed by mass spectrometry.

### 2.5. LC-ESI-MS/MS Analysis Based on Q EXACTIVE

Each fraction was resuspended in buffer A (2% acetonitrile, 0.1% FA) and centrifuged at 20 000 ×g for 10 min. In each fraction, the final concentration of peptide was about 0.5 *μ*g/*μ*L. 10 *μ*L supernatant was loaded on a LC-20AD nano-HPLC (Shimadzu, Kyoto, Japan) by the autosampler onto a 2 cm C18 trap column. Then, the peptides were eluted onto a 10 cm analytical C18 column (inner diameter 75 *μ*m) packed in-house. The samples were loaded at 8 *μ*L/min for 4 min; then the 44 min gradient was run at 300 nL/min starting from 2 to 35% B (98% acetonitrile, 0.1% FA), followed by 2-minute linear gradient to 80%, maintenance at 80% B for 4 min. Initial chromatographic conditions were restored in 1 min.

Data acquisition was performed with tandem mass spectrometry (MS/MS) in a Q EXACTIVE (Thermo Fisher Scientific, San Jose, CA) coupled online to the HPLC. Intact peptides were detected in the Orbitrap at a resolution of 70 000. Peptides were selected for MS/MS using high-energy collision dissociation (HCD) operating mode with a normalized collision energy setting of 27.0; ion fragments were detected in the Orbitrap at a resolution of 17500. In the octopole collision cell, the ten most intense peptide ions (charge states ≥ 2) were sequentially isolated to a maximum target value of 5 × 10^5^ by pAGC and fragmented HCD. A data-dependent procedure that alternated between one MS scan and 15 MS/MS scans was applied for the 15 most abundant precursor ions above a threshold ion count of 20000 in the MS survey scan with a following Dynamic Exclusion duration of 15 s. The electrospray voltage applied was 1.6 kV. Automatic gain control (AGC) was used to optimize the spectra generated by the Orbitrap. A sweeping collision energy setting of 35 ± 5 eV was applied to all precursor ions for collision-induced dissociation. The AGC target for full MS was 3e^6^ and 1e^5^ for MS^2^. For MS scans, the* m/z* scan range was 350 to 2000 Da. For MS^2^ scans, the* m/z* scan range was 100–1800 Da. The iTRAQ experiments were performed as three technical replicates to gather reliable quantitative information.

### 2.6. Data Analysis

Raw data files acquired from the Orbitrap were converted into MGF files using Proteome Discoverer 1.2 (PD 1.2, Thermo) [5600 msconverter] and the MGF files were searched. Protein identifications were performed by using Mascot search engine (Matrix Science, London, UK; version 2.3.02) against database containing 143397 sequences.

For protein identification and quantification, a peptide mass tolerance of 20 ppm was allowed for intact peptide masses and 0.05 Da for fragmented ions, with allowance for one missed cleavage in the trypsin digests. Carbamidomethylation of cysteine was considered a fixed modification, and the conversion of* N*-terminal glutamine to pyroglutamic acid and methionine oxidation were considered variable modifications. All identified peptides had an ion score above the Mascot peptide identity threshold, and a protein was considered identified if at least one such unique peptide match was apparent for the protein. To reduce the probability of false peptide identification, only peptides at the 95% confidence interval by a Mascot probability analysis greater than “identity” were counted as identified. The quantitative protein ratios were weighted and normalized by the median ratio in Mascot. We set a 1.2-fold change as the threshold and a *p* value must be below 0.05 to identify significant changes.

### 2.7. Function Method Description

Functional annotations of the proteins were conducted using Blast2 GO program against the nonredundant protein database (NR; NCBI). The KEGG database (http://www.genome.jp/kegg/) and the COG database (http://www.ncbi.nlm.nih.gov/COG/) were used to classify and group these identified proteins.

GO is an international standardization of gene function classification system. It provides a set of dynamic updating controlled vocabulary to describe genes and gene products attributes in the organism. GO has 3 ontologies which can describe molecular function, cellular component, and biological process, respectively.

COG is the database for protein orthologous classification. Every protein in COG is supposed to derive from a same protein ancestor.

KEGG PATHWAY is a collection of manually drawn pathway maps representing our knowledge on the molecular interaction and reaction networks. Molecules are represented as nodes, and the biological relationship between two nodes is represented as an edge (line).

### 2.8. Western Blot

Western blot analyses were performed to confirm the presence of differentially expressed proteins. After the treatment of the indicated concentration of ginsenoside F_2_ (10, 20, and 40 *μ*M) for 12 h, cells were harvested, washed with cold PBS (pH 7.4), and lysed with ice-cold lysis buffer (50 *μ*M Tris-HCl, 150 *μ*M NaCl, 1 *μ*M EGTA, 1 *μ*M EDTA, 20 *μ*M NaF, 100 *μ*M Na_3_VO_4_, 1%NP40, 1 *μ*M PMSF, 10 *μ*g/mL aprotinin, and 10 *μ*g/mL leupeptin, pH 7.4) for 30 min and centrifuged at 12 000 ×g for 30 min at 4°C. The protein concentration of the clear supernatant was quantified using Bio-Rad Protein Assay Kit.

Approximately 30 *μ*g of protein was loaded into a 10–15% sodium dodecyl sulfate polyacrylamide gel electrophoresis (SDS–PAGE). Thereafter, proteins were electrophoretically transferred to nitrocellulose membrane and nonspecific sites were blocked with 5% skimmed milk in 1% Tween-20 (Sigma-Aldrich) in 20 *μ*M TBS (pH 7.5) and reacted with a primary polyclonal antibody, PRR5, CISD2, Bcl-2L, NLRX1, RPS15, RPL26, p53, Atg5, Atg7, Atg10, LC3-II, LC3-I PUMA, Beclin-1, UVRAG, and mTOR and *β*-actin for 4 h at room temperature. After washing with TBS three times (5 min each), the membrane was then incubated with alkaline phosphatase-conjugated goat anti-rabbit secondary antibody. The signal was observed and developed with Kodak film by exposure to enhanced chemiluminescence (ECL) plus western Blotting Detection Reagents (Amersham Biosciences, Piscataway, NJ, USA).

### 2.9. Statistical Analysis

For cell-based assay, experiments were performed in duplicate and three independent experiments were performed. Western blot analyses of differential protein expressions were validated on cell lysates from three biological replicates. Statistical significance was analyzed using Student's* t*-test or ANOVA test by using GraphPad Prism v4.0 software (GraphPad Software, San Diego, CA, USA). Statistical significance is expressed as ^*∗∗∗*^ *p* < 0.001; ^*∗∗*^ *p* < 0.01; ^*∗*^ *p* < 0.05.

## 3. Results 

### 3.1. Proteome Analysis

Human gastric carcinoma cells (SGC7901) are treated with ginsenoside F_2_ at a dose of 20 *μ*M for 12 hours. The harvested proteins are used to perform iTRAQ for quantifying the difference of total 31853 peptides and 5411 proteins in SGC7901 cells with or without treatment. Finally, 205 proteins were screened out in terms of the change in their expression level which meet our predefined criteria of *p* < 0.05 with relative expression levels at least >1.2-fold (Table 1) or <0.83-fold (Table 2) (both 113/115 and 113/117) in ginsenoside F_2_-treated group compared with the control group. The protein properties, including pI, molecular weight (MW), and number of residues were calculated by Mascot. The results are highly reproducible in two individual experiments.

### 3.2. Classification of Differentially Expressed Proteins

**Figure 2:**
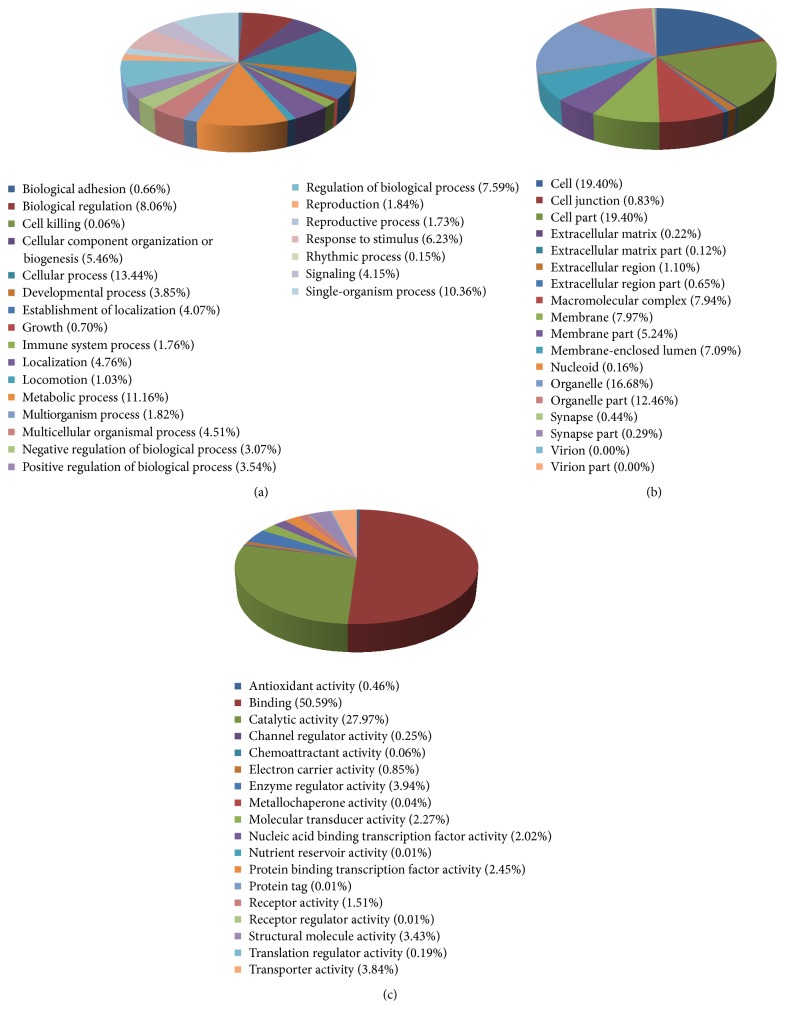
Classification of identified proteins. (a) The biological processes (BPs), (b) cellular components (CCs), and (c) molecular functions (MFs) of the total identified proteins classified by GO database.

**Figure 3:**
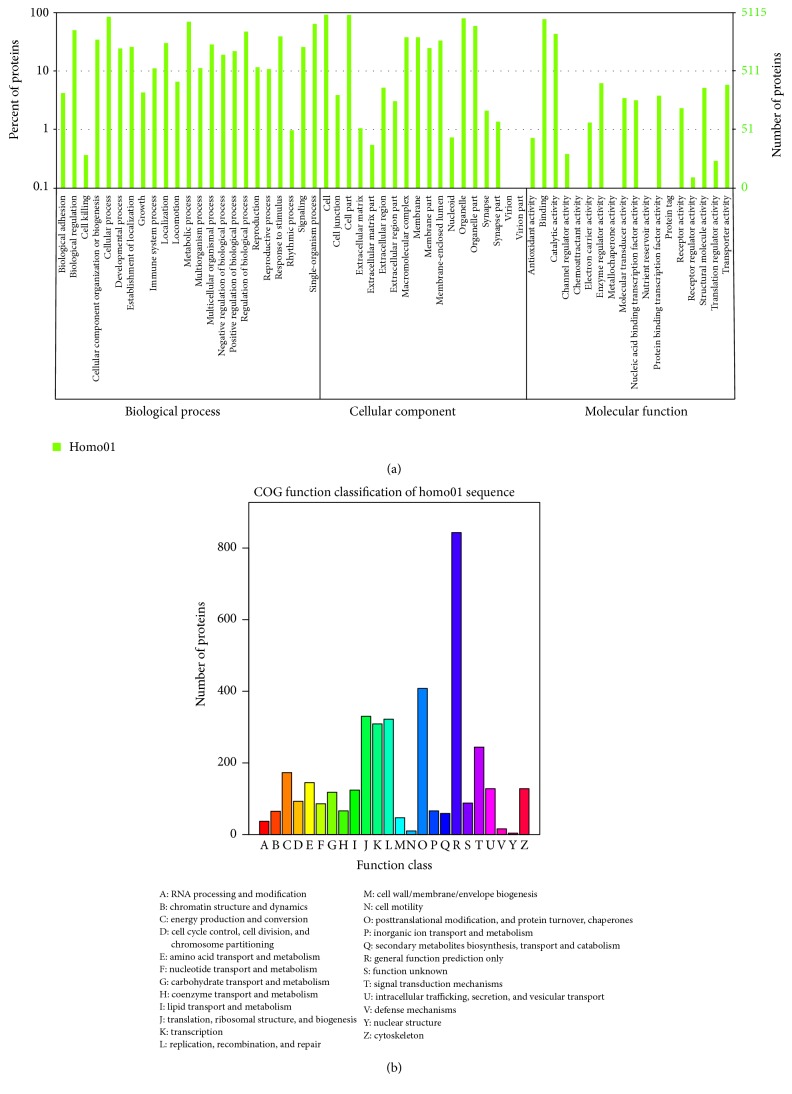
WEGO (a) and COG (b) assay of the 205 differentially expressed proteins.

Firstly, screened proteins were functionally catalogued with GO and WEGO to three different groups (Figures 2 and 3(a)): biological process (BP), cellular component (CC), and molecular function (MF). As shown in Figure 2, the proteins are involved in BP including cellular process (13.44%), metabolic process (11.16%), single-organism process (10.36%), biological regulation (8.06%), and regulation of biological process (7.59%). The identified proteins separated according to CC include cell (19.40%), cell part (19.40%), organelle (16.68%), organelle part (12.46%), membrane (7.97%), and macromolecular complex (7.94%). MF of the proteins was classified and large groups were found to be binding (50.59%), catalytic activity (27.97%), enzyme regulator activity (3.94%), transporter activity (3.84%), and structural molecular activity (3.43%).

**Figure 4:**
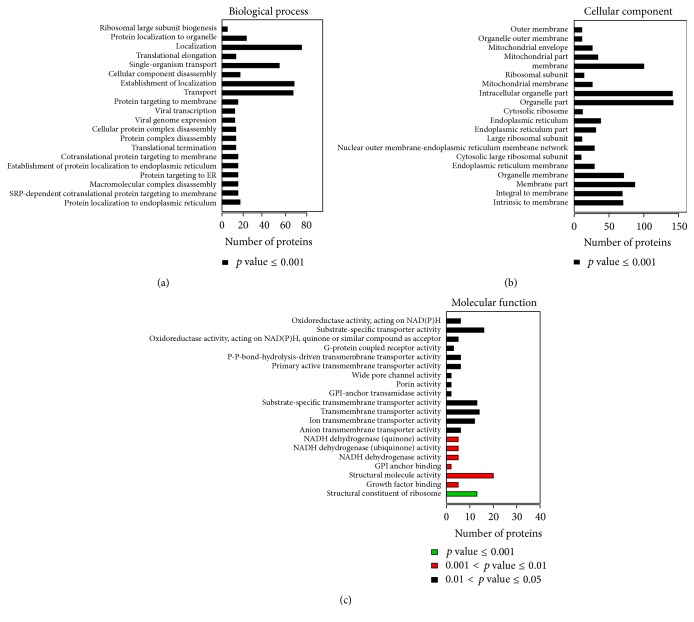
GO annotation of the final selected differentially expressed proteins. The top 20 components for BP (a), CC (b), and MF (c) of the selected differentially expressed proteins are shown along with their enrichment score, represented as a *p* value.

Further COG function classification revealed that posttranslational modification, protein turnover, and ribosomal structure biogenesis were major function of the screened 205 proteins (Figure 3(b)). In each category of BP, CC, and MF, top twenty proteins which generated bigger difference in response to ginsenoside F_2_ treatment are listed in Figure 4.

**Figure 5:**
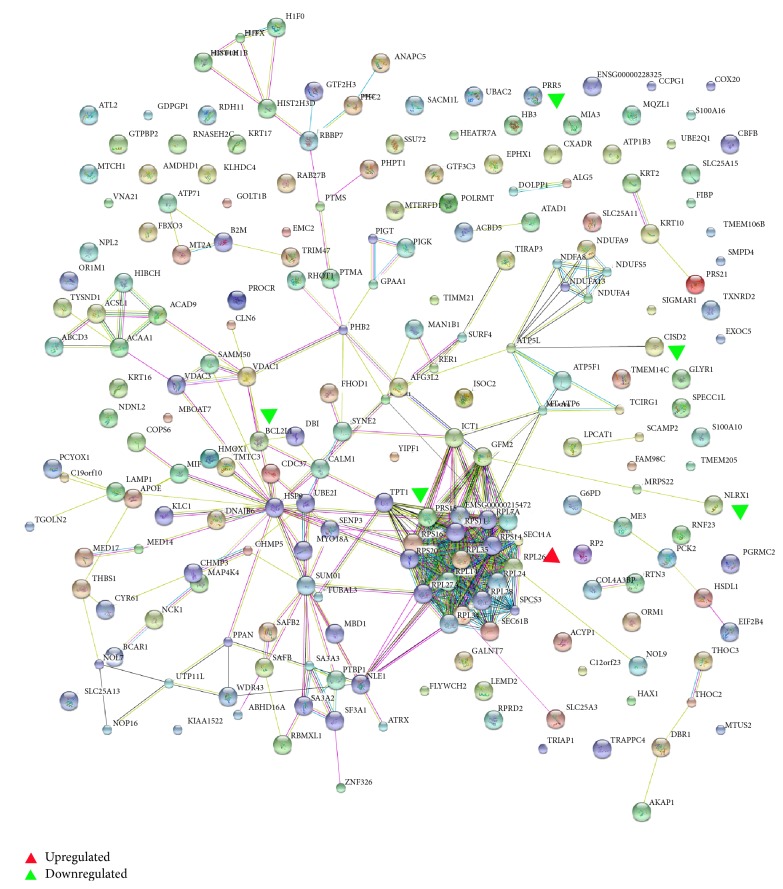
The protein-protein interaction network of the differentially expressed proteins identified. Red triangle denotes upregulated proteins; green triangle denotes downregulated protein.

KEGG is a publicly available pathway database and could provide biologists excellent resources to attain a deeper understanding of biological mechanisms in response to different treatments. Protein analysis through KEGG indicated that 205 differentially expressed proteins were involved in 128 different pathways (data not shown). The connection degree between proteins is calculated by protein-protein interaction network analysis and the results are shown in Figure 5. Among these proteins, PRR5, RPS15, and RPL26 were found in ribosomal protein signaling pathway; CISD2, Bcl-xl, and NLRX1 were found in Beclin-1/Bcl-xL pathway. Therefore, PRR5, RPS15, RPL26, CISD2, Bcl-xl, and NLRX1 were selected for further validation and study in order to provide a comprehensive perspective for elucidating underlying molecular mechanisms of ginsenoside F_2_.

### 3.3. Western Blot Analysis

#### 3.3.1. For Verification

**Figure 6:**
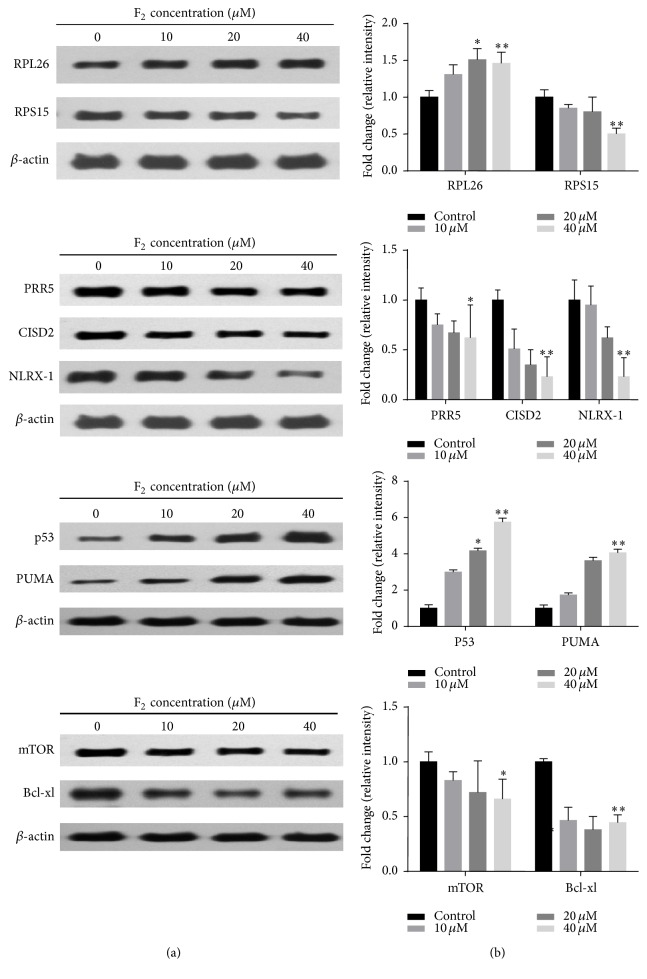
Western blot validations of RPS15, RPL26, PRR5, CISD2, NLRX1, p53, PUMA, mTOR, and Bcl-xl in SGC7901 cells with different concentrations of ginsenoside F_2_. 1 × 10^6^ SGC7901 cells are seeded in 6-well plate for overnight. On day 2, the cultured cells are treated with different concentration ginsenoside F_2_. 12 hours after treatment, the protein is prepared by lysating cells with RIPA buffer for performing western blot analysis. Left panel: the representative western blot analysis. *β*-actin was used as the loading control. Right panel: accumulated results show the relative protein density. Error bars represent means ± SEMs. Significant difference is expressed as ^*∗∗*^ *p* < 0.01, ^*∗*^ *p* < 0.05.

To validate the information obtained from the iTRAQ-based quantitative proteomics study and bioinformatics analysis, the screened proteins with strong response to ginsenoside F_2_ treatment were further confirmed by western blot. As shown in Figure 6, ginsenoside F_2_ significantly reduced protein expressions of PRR5, CISD2, Bcl-xl, NLRX1, and RPS15 (*p* < 0.01) and enhanced the expression of the RPL26 (*p* < 0.01) in SGC7901 cells in comparison with the treatment with vehicle control.

#### 3.3.2. For Determining the Expression of Apoptosis and Autophagic Proteins

**Figure 7:**
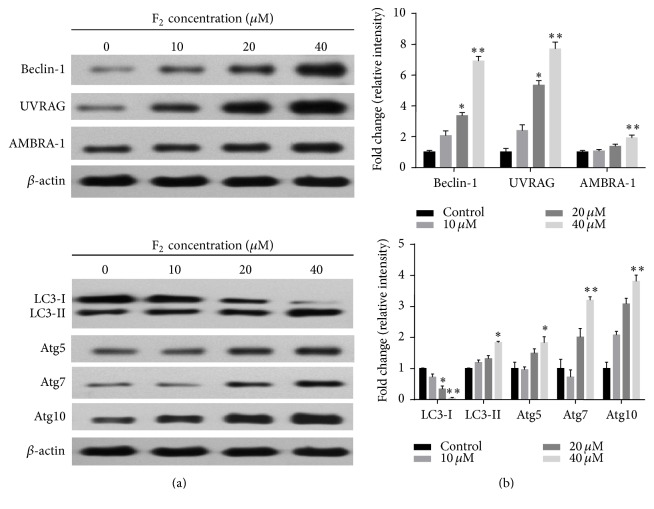
Effect of ginsenoside F_2_ on the expression of Beclin-1, UVRAG, AMBRA-1, Atg5, Atg7, Atg10, LC3 I, and LC3-II. 1 × 10^6^ SGC7901 cells are seeded in 6-well plate for overnight. On day 2, the cultured cells are treated with different concentration ginsenoside F_2_. 12 hours after treatment, the protein is prepared by lysating cells with RIPA buffer for performing western blot analysis. Left panel: the representative western blot analysis. *β*-actin was used as the loading control. Right panel: accumulated results show the relative protein density. Error bars represent means ± SEMs. Significant difference is expressed as ^*∗∗*^ *p* < 0.01, ^*∗*^ *p* < 0.05.

As shown in Figure 6, ginsenoside F_2_ suppressed the expression of mTOR and upregulated the expression of p53 in a dose-dependent manner. Atg5, Atg7, Atg10, PUMA, Beclin-1, UVRAG, and AMBRA-1 are known to be modulated by p53 or Bcl-xl signaling, which may trigger apoptosis or autophagy. Therefore, we proceeded to check the expressions of Atg5, Atg7, Atg10, PUMA, Beclin-1, UVRAG, and AMBRA-1. As shown in Figure 7, ginsenoside F_2_ upregulated the expressions of these proteins in a dose-dependent manner. LC3 is now widely used to monitor autophagy. During autophagy, the cytoplasmic form LC3-I is processed and recruited to phagophores, where LC3-II is generated by site-specific proteolysis and lipidation at the C-terminus. Thus, the amount of LC3-II positively correlates with the number of autophagosomes [10]. We examined the effect of F_2_ on LC3 conversion in SGC7901 cells. Western blot analysis showed that F_2_ treatment resulted in dose-dependent accumulation of LC3-II and reduction of LC3-I (Figure 7). The conversion of LC3-I to LC3-II suggested F_2_ treatment induces autophagy.

In the present study, combination of iTRAQ-based proteomics method with bioinformatics was used to identify critical molecules in SGC7901 cancer cells in response to ginsenoside F_2_ treatment. Ginsenoside F_2_ generated significant change of protein profile in SGC7901 cells. Some of them have been demonstrated to participate in either apoptosis or autophagy responses, suggesting that the antitumor mechanisms of ginsenoside F_2_ in SGC7901 cells are involved in both apoptosis and autophagy.

The current findings demonstrate that ginsenoside F_2_ impacts distinct signaling pathways and induces broad change in the protein profile of SGC7901 cells. Overall, 205 differentially expressed proteins were identified with ≥95% confidence in ginsenoside F_2_ treated group. Application of a ratio of 1.2-fold change as criteria resulted in 44 and 161 differentially abundant proteins in SGC7901 cells.

In our study, some proteins that were significantly altered by ginsenoside F_2_ show close relationship of protein-protein interaction (Figure 5). Ribosomal proteins, such as RPS15 and RPL26, exert critical roles in MDM2-p53 signal pathway [11, 12]. PRR5 [13], CISD2 [14], Bcl-xl [15], and NLRX1 [16, 17] have been reported to play a key role in the regulation of autophagy or apoptosis. The changes of these six potential proteins were verified by western blot analysis.

Ribosomal proteins (RPs) are considered to have diverse extra ribosomal functions, ranging from cell cycle progression to cell death and to malignant transformation and cellular metabolism [11]. Relevantly, a number of RPs have been shown to bind to MDM2, the inhibitor of p53 (murine double minute 2, and also HDM2 for its human ortholog), and inhibit MDM2 E3 ligase activity, leading to p53 stabilization and activation, then triggering apoptosis or autophagy [11]. Following the treatment of ginsenoside F_2_ in SGC7901 cells, the levels of RPL28, RPL34, RPL35, RPS16, RPL17, RPL14, RPL24, RPL7A, and RPL26 were increased, whereas that of RPS15 reduced. Although the functions of RPL28, RPL34, RPL35, RPS16, RPL17, RPL14, RPL24, and RPL7A have not been well studied, RPL26, a positive regulator of p53, was found to increase the translational rate of p53 mRNA by binding to its 50 untranslated region [12] and, in this case, MDM2 acts as an ubiquitin E3 ligase for ubiquitylation and degradation of RPL26 [18]. Thus, under the treatment of ginsenoside F_2_, the increased level of RPL26 indicated that RPL26 may inhibit MDM2 and subsequently activate p53. RPS15, identified as a direct p53 transcriptional target, was thought to activate p53 by repressing MDM2 activity [19]. Interestingly, in our study, the level of RPS15 reduced in SGC7901 followed by ginsenoside F_2_ treatment, suggesting that the roles of RPS15 and RPL26 involved in the anticancer mechanism of ginsenoside F_2_ are different, which warrant further investigation.

mTOR, existing in two multiprotein complexes, mTORC1 and mTORC2, regulates cell growth in response to a variety of cellular signals derived from growth factors and environmental stress [20]. mTORC2 is a kinase complex comprised of mTOR, PRR5, Rictor, mSin1, and mLST8/GbL. The expression level of PRR5 is correlated with that of mTORC2. Recent study showed that mTORC2 is implicated in actin cytoskeleton regulation, as well as phosphorylation of Akt [13]. Although TOR kinase has been largely attributed as a negative regulator of autophagy through TORC1, resent study indicated that mTORC2 was an independent positive regulator of autophagy during amino acid starvation [21]. In the present study, ginsenoside F_2_ decreased level of PPR5, indicated that ginsenoside F_2_ may inhibit the expression of PRR5, and consequently inhibited mTORC2.

Recent study indicated that p53 can be a positive or negative regulator of autophagy. In the nucleus, p53 may activate the AMPK pathway and inhibit the mTOR pathway, subsequently triggering autophagy. p53 may also transactivate multiple genes with proautophagic roles, including proapoptotic Bcl-2 proteins (Bax, PUMA) [22, 23]. In this network, PUMA induces the noncanonical autophagy pathway regulated via Atg5, Atg7, and Atg10. PUMA's initiation of autophagy promotes cytochrome c release, which then leads to apoptosis [22]. Interestingly, in our previous work, increasing level of cytochrome c and decreased mitochondrial transmembrane potential (MTP) were observed [6]. In present study, decreased expressions of PRR5 and RPL26 were found, which implied that ginsenoside F_2_ might trigger p53 signal pathway. It was reported that western blot analyses tended to show greater differential abundance compared with iTRAQ analyses [24]. Thus, the expressions of p53, Atg5, Atg7, Atg10, and PUMA were validated by western blot analyses. The increased level of Atg5 Atg7, Atg10, and PUMA and reduced level of P53 and mTORC2 suggested that ginsenoside F_2_ may initiate autophagy by ribosomal protein-p53 signaling pathway.

CISD2, also known as NAF-1, Miner1, Eris, and Noxp70, is a member of the 2Fe-2S cluster NEET family [25]. Our results showed that CISD2 was significantly decreased in ginsenoside F_2_ treated group, confirmed by western blot analysis. Recent work identified CISD2 as a Bcl-xl binding partner at a branch point between autophagy and apoptosis, life and death, under nutrient-deprived and oxidative stress conditions* in vivo* cells [25, 26]. Bcl-xl, also called Bcl-2L, is known to function through inhibition of the autophagy effector and tumor suppressor Beclin-1 [15]. CISD2 is required in this pathway for Bcl-xl to functionally antagonize Beclin-1-dependent autophagy. In our study, the expression of Bcl-xl decreased, confirmed by western blot analysis. Thus, CISD2 may be a Bcl-xl-associated cofactor that targets Bcl-2 for the autophagy pathway.

During initiation of autophagosome formation, after release from Bcl-xl, Beclin-1 functions as a platform by binding to class III PI3K/vacuolar protein sorting-34 (Vps34), UV-resistance-associated gene (UVRAG), activating molecule in Beclin-1-regulated autophagy (AMBRA-1) [15, 26, 27]. Previous studies have shown that binding of Beclin-1 to Bcl-2/Bcl-xl inhibits the autophagic function of Beclin-1, suggesting that Beclin-1 might have a role in the convergence between autophagy and apoptotic cell death [22]. For confirming the Beclin-1/Bcl-xl pathway, western blot was employed. The expressions of Beclin-1, UVRAG, and AMBRA-1 were increased, while Bcl-xl was decreased, which suggested that ginsenoside F_2_ may induce autophagy via Bcl-xl/Beclin-1 pathway.

NLRX1, a mitochondrial NOD-like receptor that amplifies apoptosis by inducing reactive oxygen species production, is an important component of TLR mediated inflammatory pathways [13, 16]. Recent evidence suggested that upregulated expression of NLRX1 may synergistically regulate metabolism and autophagy for highly invasive growth of the autophagy addicted MDA-MB-231 breast cancer cells [16]. And it acted as tumor suppressor by regulating TNF-*α* induced apoptosis and metabolism in cancer cells. In our iTRAQ results, expression of NLRX1 was significantly decreased in SGC7901 cells treated with ginsenoside F_2_. The phenomenon suggested different role of NLRX1 involved in the ginsenoside F_2_ treatment that may be different from that of published reports [16, 17], though the mechanism needs further research.

Mai et al. reported that F_2_ induces apoptotic cell death accompanied by protective autophagy in breast cancer stem cells [28]. In one of our previous studies, we found that F_2_ induces apoptosis by causing an accumulation of ROS and activating the apoptosis signaling pathway [6]. However, there was no report systemically comparing differently regulated proteins and building a network of F_2_-treated cancer cells at proteome level. In the current study, by the close look at cellular mechanisms at proteome level, we clearly identified the distinct pattern of cellular responses for the F_2_-treated cells, and 6 differentially regulated proteins were identified, which provide useful information on elucidating the anticancer mechanism of F_2_ to SGC7901 cells. Moreover, the integration of networks and pathway with the proteomic data enhanced our understanding of the functional relationship of proteome changes caused by the compound.

## 4. Conclusions

In conclusion, 44 upregulated proteins and 161 downregulated proteins were discovered by iTRAQ analysis in SGC7901 cells treated with lower dose and shorter duration of ginsenoside F_2_, compared with our previous study. 6 differentially abundant common proteins, PRR5, CISD2, Bcl-xl, NLRX1, RPS15, and RPL26, were confirmed by western blot analysis. Ribosomal protein-p53 signaling pathway and Bcl-xl/Beclin-1 pathway might be significantly regulated biological process by ginsenoside F_2_ treatment in SGC7901 cells. Although more work is required to find out the precise role of targeted proteins, our data lead to a better understanding of the molecular mechanisms of ginsenoside F_2_ for gastric cancer treatment.

## Figures and Tables

**Table 1:** Differentially upregulated (>1.20-fold) proteins identified by iTRAQ in F_2_ treated SGC7901 cells.

Rank #	Accession	Gene symbol (GN)	Definition (description)	Score	Mass	Cov%	Ration	COG function-description
Up 1	sp|P07305-2	H1F0	Isoform 2 of histone H1.0	51	35582	13	2.11	—
Up 2	sp|P20962	PTMS	Parathymosin	503	15782	23.5	1.32	—
Up 3	tr|B8ZWD1	DBI	Diazepam binding inhibitor, splice form 1A(2)	121	15706	28.9	1.31	Acyl-CoA-binding protein
Up 4	sp|Q16576	RBBP7	Histone-binding protein RBBP7	877	55737	24.5	1.25	FOG: WD40 repeat
Up 5	sp|P46779-2	RPL28	Isoform 2 of 60S ribosomal protein L28	524	22107	27.6	1.35	—
Up 6	tr|B2R514	—	cDNA, FLJ92300, Homo sapiens COP9 subunit 6 (MOV34 homolog, 34 kD) (COPS6), mRNA	74	39068	20.2	1.22	Predicted metal-dependent protease of the PAD1/JAB1 superfamily
Up 7	tr|B3KY12	—	cDNA FLJ46581 fis, clone THYMU3043200, highly similar to splicing factor 3A subunit 3	527	71859	22	1.24	Splicing factor 3a, subunit 3
Up 8	sp|Q71DI3	HIST2H3A	Histone H3.2	617	19694	26.5	1.40	Histones H3 and H4
Up 9	tr|Q9P0H9	RER1	RER1 protein	118	28927	22	1.26	Golgi protein involved in Golgi-to-ER retrieval
Up 10	tr|A8K3Q9	—	cDNA FLJ76611, highly similar to Homo sapiens ribosomal protein L14 (RPL14), mRNA	781	35114	25.9	2.24	Ribosomal protein L14E/L6E/L27E
Up 11	sp|Q9Y3A2	UTP11L	Probable U3 small nucleolar RNA-associated protein 11	94	44174	21.7	1.30	Uncharacterized conserved protein
Up 12	tr|F2Z388	RPL35	60S ribosomal protein L35	99	15372	32.3	1.35	Ribosomal protein L29
Up 13	sp|Q9NZZ3	CHMP5	Charged multivesicular body protein 5	268	32218	21	1.42	—
Up 14	tr|B2R4D8	—	60S ribosomal protein L27	398	23061	36	1.28	Ribosomal protein L14E/L6E/L27E
Up 15	tr|M0QXF7	C19orf10	UPF0556 protein C19orf10 (fragment)	265	11851	25	1.24	—
Up 16	tr|D3DV26	S100A10	S100 calcium binding protein A10 (annexin II ligand, calpactin I, light polypeptide (P11)), isoform CRA_b (fragment)	134	27935	8.3	1.21	—
Up 17	tr|H7C2N1	PTMA	Thymosin alpha-1 (fragment)	117	18283	8.8	1.30	—
Up 18	tr|G2XKQ0	—	Sumo13	60	14938	11.9	1.22	Ubiquitin-like protein (sentrin)
Up 19	tr|I3L1Y9	FLYWCH2	FLYWCH family member 2	99	19302	47.2	1.45	—
Up 20	tr|M0R210	RPS16	40S ribosomal protein S16	1105	19391	57.4	1.27	Ribosomal protein S9
Up 21	sp|O43715	TRIAP1	TP53-regulated inhibitor of apoptosis 1	82	12050	18.4	1.36	—
Up 22	sp|P49207	RPL34	60S ribosomal protein L34	187	18684	20.5	1.66	Ribosomal protein L34E
Up 23	sp|Q92522	H1FX	Histone H1x	342	35250	25.4	1.33	—
Up 24	tr|J3KRX5	RPL17	60S ribosomal protein L17 (fragment)	795	27382	38.5	1.26	Ribosomal protein L22
Up 25	sp|P02795	MT2A	Metallothionein-2	104	9915	52.5	1.42	—
Up 26	tr|Q6FIE5	PHP14	PHP14 protein	72	17301	8.8	1.27	—
Up 27	tr|A0PJ62	RPL14	RPL14 protein (fragment)	536	21409	43.5	2.85	Ribosomal protein L14E/L6E/L27E
Up 28	tr|G3XAA2	MAP4K4	Mitogen-activated protein kinase kinase kinase kinase 4	142	156989	2.7	1.24	Serine/threonine protein kinase
Up 29	tr|C9JNW5	RPL24	60S ribosomal protein L24	666	24642	32	1.67	Ribosomal protein L24E
Up 30	sp|Q13951	CBFB	Core-binding factor subunit beta	197	24461	18.1	1.20	—
Up 31	tr|D3DUE6	N-PAC	Cytokine-like nuclear factor n-pac, isoform CRA_c	219	76728	14.5	1.24	3-Hydroxyisobutyrate dehydrogenase and related beta-hydroxy acid dehydrogenases
Up 32	tr|K7EKW4	ISOC2	Isochorismatase domain-containing protein 2, mitochondrial (fragment)	130	21202	17.4	1.34	Amidases related to nicotinamidase
Up 33	sp|Q9NQ55-2	PPAN	Isoform 2 of Suppressor of SWI4 1 homolog	73	63713	10.7	1.37	—
Up 34	tr|B3KMF8	—	cDNA FLJ10869 fis, clone NT2RP4001677	127	12398	27.7	1.28	—
Up 35	sp|P62424	RPL7A	60S ribosomal protein L7a	613	42316	27.1	1.78	Ribosomal protein HS6-type (S12/L30/L7a)
Up 36	tr|B4E0X1	—	Beta-2-microglobulin	185	17093	13.1	1.25	—
Up 37	tr|H0Y7A7	CALM2	Calmodulin (fragment)	735	24209	30.5	1.26	Ca^2+^-binding protein (EF-Hand superfamily)
Up 38	tr|J3KTJ8	RPL26	60S ribosomal protein L26 (fragment)	363	15545	34	1.24	Ribosomal protein L24
Up 39	tr|B4DJM5	—	cDNA FLJ61294, highly similar to keratin, type I cytoskeletal 17	326	21291	24.9	1.46	—
Up 40	sp|Q9Y3C1	NOP16	Nucleolar protein 16	79	27925	20.8	1.24	—
Up 41	sp|Q16543	CDC37	Hsp90 cochaperone Cdc37	384	57730	29.6	1.22	—
Up 42	sp|P16401	HIST1H1B	Histone H1.5	801	42644	17.3	2.38	—
Up 43	sp|Q07866-3	KLC1	Isoform G of kinesin light chain 1	642	81828	23.9	1.24	FOG: TPR repeat
Up 44	tr|B4DKJ4	—	cDNA FLJ57738, highly similar to translationally controlled tumor protein	344	19250	32.4	1.28	—

**Table 2:** Differentially downregulated (<0.83-fold) proteins identified by iTRAQ in F_2_ treated SGC7901 cells.

Rank #	Accession	Gene symbol (GN)	Definition (description)	Score	Mass	Cov%	Ration	COG function-description
Down 1	tr|F5H740	VDAC3	Voltage-dependent anion-selective channel protein 3	1114	39598	41.5	0.81	—
Down 2	sp|Q9H845	ACAD9	Acyl-CoA dehydrogenase family member 9, mitochondrial	311	81512	21.9	0.69	Acyl-CoA dehydrogenases
Down 3	sp|Q969S9-2	GFM2	Isoform 2 of ribosome-releasing factor 2, mitochondrial	153	94059	5.1	0.80	Translation elongation factors (GTPases)
Down 4	sp|P35908	KRT2	Keratin, type II cytoskeletal 2 epidermal	338	76630	18.2	0.67	Myosin heavy chain
Down 5	tr|B7Z8A2	—	cDNA FLJ51671, highly similar to prenylcysteine oxidase (EC 1.8.3.5)	492	63740	23.8	0.83	—
Down 6	sp|Q9Y512	SAMM50	Sorting and assembly machinery component 50 homolog	170	59339	18.6	0.76	Outer membrane protein/protective antigen OMA87
Down 7	sp|Q6ZNW5	GDPGP1	GDP-D-glucose phosphorylase 1	118	45302	8.6	0.78	—
Down 8	sp|P51970	NDUFA8	NADH dehydrogenase [ubiquinone] 1 alpha subcomplex subunit 8	72	25720	15.1	0.68	—
Down 9	tr|B4DRW0	—	cDNA FLJ58125, highly similar to copper-transporting ATPase 1 (EC 3.6.3.4)	102	61873	6.1	0.78	Cation transport ATPase
Down 10	tr|Q8NBW7	KDELR1	ER lumen protein retaining receptor	51	20327	12.7	0.73	ER lumen protein retaining receptor
Down 11	tr|B2R6F5	—	cDNA, FLJ92928, highly similar to Homo sapiens retinitis pigmentosa 2 (X-linked recessive) (RP2), mRNA	59	47451	2.3	0.82	—
Down 12	tr|Q2VIN3	—	RBM1 (fragment)	1232	45756	26.8	0.81	RNA-binding proteins (RRM domain)
Down 13	sp|P14174	—	Macrophage migration inhibitory factor	608	13856	17.4	0.71	—
Down 14	tr|B2R6S4	—	cDNA, FLJ93089, highly similar to Homo sapiens NCK adaptor protein 1 (NCK1), mRNA	137	53755	18.3	0.83	—
Down 15	sp|Q16822	PCK2	Phosphoenolpyruvate carboxykinase [GTP], mitochondrial	1795	78784	41.6	0.74	Phosphoenolpyruvate carboxykinase (GTP)
Down 16	tr|E9PM12	TCIRG1	V-type proton ATPase 116 kDa subunit a isoform 3 (fragment)	63	25815	13.3	0.74	Archaeal/vacuolar-type H^+^-ATPase subunit I
Down 17	sp|Q2T9J0-2	TYSND1	Isoform 2 of peroxisomal leader peptide-processing protease	96	43618	9.8	0.67	—
Down 18	tr|J3KPX7	PHB2	Prohibitin-2	1543	39466	51.8	0.82	Membrane protease subunits, stomatin/prohibitin homologs
Down 19	tr|Q8NCF7	—	cDNA FLJ90278 fis, clone NT2RP1000325, highly similar to phosphate carrier protein, mitochondrial precursor	517	48576	26.9	0.81	—
Down 20	tr|B4E0R0	—	cDNA FLJ54220, highly similar to Long-chain-fatty-acid-CoA ligase 1 (EC 6.2.1.3)	100	88560	6.2	0.74	Long-chain acyl-CoA synthetases (AMP-forming)
Down 21	tr|B3KRY3	—	cDNA FLJ35079 fis, clone PLACE6005283, highly similar to lysosome-associated membrane glycoprotein 1	319	48851	11.1	0.79	—
Down 22	tr|B3KU09	—	cDNA FLJ39034 fis, clone NT2RP7008085, highly similar to Homo sapiens ring finger protein 123 (RNF123), mRNA	110	166029	2.4	0.78	—
Down 23	sp|Q9BVV7	TIMM21	Mitochondrial import inner membrane translocase subunit Tim21	86	35219	13.7	0.82	—
Down 24	sp|Q9UMY1	NOL7	Nucleolar protein 7	148	39504	12.5	0.78	—
Down 25	sp|Q9UNN8	PROCR	Endothelial protein C receptor	103	27909	15.1	0.80	—
Down 26	sp|Q86SF2	GALNT7	N-Acetylgalactosaminyltransferase 7	95	89410	9.9	0.81	—
Down 27	tr|I3L0U2	PRSS21	Testisin (fragment)	115	27083	14.7	0.82	Secreted trypsin-like serine protease
Down 28	tr|B7ZLP5	SAFB	SAFB protein	557	121835	13	0.83	—
Down 29	tr|F2Z3N7	TMEM106B	Transmembrane protein 106B	135	12975	12.5	0.82	—
Down 30	tr|B7Z361	—	Reticulon	166	27838	12.2	0.76	—
Down 31	tr|H0Y6F2	PRR5	Proline-rich protein 5 (fragment)	57	39929	2.3	0.78	—
Down 32	sp|Q7Z7E8	UBE2Q1	Ubiquitin-conjugating enzyme E2 Q1	92	54711	1.9	0.76	—
Down 33	tr|A8K4K9	—	cDNA FLJ76169	146	42007	8.8	0.83	—
Down 34	sp|P13645	KRT10	Keratin, type I cytoskeletal 10	382	66321	21.6	0.55	—
Down 35	sp|Q8N5K1	CISD2	CDGSH iron-sulfur domain-containing protein 2	167	20364	26.7	0.81	—
Down 36	sp|Q8NI27	THOC2	THO complex subunit 2	282	241732	8.7	0.83	—
Down 37	tr|B4DEP8	—	cDNA FLJ56960, highly similar to Homo sapiens phosphatidylinositol 4-kinase type II (PI4KII), mRNA	127	61711	9.8	0.76	—
Down 38	sp|Q5BKZ1	ZNF326	DBIRD complex subunit ZNF326	145	78123	7.9	0.78	—
Down 39	tr|Q8IW24	EXOC5	Exocyst complex component 5	108	99962	9.3	0.82	—
Down 40	tr|B3KMG6	—	cDNA FLJ10939 fis, clone OVARC1001065, highly similar to Homo sapiens MTERF domain containing 1 (MTERFD1), mRNA	117	43225	9.8	0.76	—
Down 41	sp|Q8NBM4-2	UBAC2	Isoform 2 of ubiquitin-associated domain-containing protein 2	150	37306	18.1	0.83	—
Down 42	sp|Q8NGA1	OR1M1	Olfactory receptor 1M1	76	39512	2.2	0.69	—
Down 43	tr|E9PN17	ATP5L	ATP synthase subunit g, mitochondrial	366	11489	63.2	0.82	—
Down 44	tr|B2R686	TGOLN2	Trans-golgi network protein 2, isoform CRA_a	166	61093	13	0.79	—
Down 45	tr|B4DIR5	—	cDNA FLJ56026	51	143728	1.7	0.74	—
Down 46	tr|J3KS15	ICT1	Peptidyl-tRNA hydrolase ICT1, mitochondrial (fragment)	169	26740	26	0.82	Protein chain release factor B
Down 47	tr|F5H0F9	ANAPC5	Anaphase-promoting complex subunit 5	72	98300	7.5	0.82	—
Down 48	tr|C8C504	HBB	Beta-globin	1233	20056	29.9	0.21	—
Down 49	tr|B2R921	—	cDNA, FLJ94171, highly similar to Homo sapiens solute carrier family 25 (mitochondrial carrier; ornithine transporter) member 15 (SLC25A15), nuclear gene encoding mitochondrial protein, mRNA	53	39308	9	0.77	—
Down 50	sp|Q9Y613	FHOD1	FH1/FH2 domain-containing protein 1	255	141625	8.8	0.81	—
Down 51	sp|Q92643	PIGK	GPI-anchor transamidase	110	51592	10.9	0.77	Glycosylphosphatidylinositol transamidase (GPIT), subunit GPI8
Down 52	tr|A4FTY4	TXNRD2	TXNRD2 protein	331	41672	24.6	0.79	Pyruvate/2-oxoglutarate dehydrogenase complex, dihydrolipoamide dehydrogenase (E3) component, and related enzymes
Down 53	tr|D3DP46	SPCS3	Signal peptidase complex subunit 3 homolog (*S. cerevisiae*), isoform CRA_a	147	24007	18.9	0.82	—
Down 54	sp|Q9Y5Q9	GTF3C3	General transcription factor 3C polypeptide 3	154	117216	7.8	0.79	—
Down 55	sp|P60468	SEC61B	Protein transport protein Sec61 subunit beta	192	11546	37.5	0.72	—
Down 56	sp|Q5RI15-2	—	Isoform 2 of cytochrome c oxidase protein 20 homolog	106	17682	20	0.83	—
Down 57	sp|Q9P206-2	—	Isoform 2 of uncharacterized protein KIAA1522	146	128602	6.5	0.73	—
Down 58	sp|Q86YN1	DOLPP1	Dolichyldiphosphatase 1	64	28953	5.5	0.69	Membrane-associated phospholipid phosphatase
Down 59	sp|O00165-2	—	Isoform 2 of HCLS1-associated protein X-1	111	34281	16	0.81	—
Down 60	tr|B4E303	—	cDNA FLJ57449, highly similar to Notchless homolog 1	127	54134	16.5	0.82	FOG: WD40 repeat
Down 61	sp|O00194	RAB27B	Ras-related protein Rab-27B	56	29688	14.2	0.77	GTPase SAR1 and related small G proteins
Down 62	tr|B4DI41	MBD1	Methyl-CpG-binding domain protein 1	72	87409	1.8	0.80	—
Down 63	tr|B0UXB6	ABHD16A	Abhydrolase domain-containing protein 16A	129	73275	10.3	0.83	Hydrolases of the alpha/beta superfamily
Down 64	sp|Q5T8D3-2	—	Isoform 2 of Acyl-CoA-binding domain-containing protein 5	148	64353	11.6	0.72	Acyl-CoA-binding protein
Down 65	tr|B4DNZ6	GTF2H3	General transcription factor IIH subunit 3	48	37020	4.5	0.79	RNA polymerase II transcription initiation/nucleotide excision repair factor TFIIH, subunit TFB4
Down 66	sp|Q96FQ6	S100A16	Protein S100-A16	346	15197	22.3	0.83	—
Down 67	tr|B4DSE1	—	cDNA FLJ55364, highly similar to CRSP complex subunit 6	55	84524	3.7	0.73	—
Down 68	tr|J3KNX9	MYO18A	Unconventional myosin-XVIIIa	157	282257	3.5	0.72	Myosin heavy chain
Down 69	tr|B4DMK6	—	cDNA FLJ60055, highly similar to *Rattus norvegicus* Ssu72 RNA polymerase II CTD phosphatase homolog, mRNA	51	23745	13.5	0.82	RNA polymerase II-interacting protein involved in transcription start site selection
Down 70	tr|G3V1A0	TRAPPC4	HCG38438, isoform CRA_b	51	14838	20.5	0.81	—
Down 71	tr|B1AHA8	HMOX1	Heme oxygenase 1 (fragment)	53	25525	15.5	0.83	Heme oxygenase
Down 72	sp|Q9Y3B3-2	TMED7	Isoform 2 of transmembrane emp24 domain-containing protein 7	193	24908	28.2	0.82	—
Down 73	tr|G3V1U5	GOLT1B	Golgi transport 1 homolog B (*S. cerevisiae*), isoform CRA_c	167	9121	20.3	0.77	Membrane protein involved in Golgi transport
Down 74	tr|B1PBA3	—	SKNY protein	148	109440	8.4	0.81	—
Down 75	sp|Q15061	WDR43	WD repeat-containing protein 43	138	91327	5.6	0.83	FOG: WD40 repeat
Down 76	tr|D3DUJ0	AFG3L2	AFG3 ATPase family gene 3-like 2 (yeast), isoform CRA_a (fragment)	695	103842	21.2	0.83	ATP-dependent Zn proteases
Down 77	tr|B2RBL9	—	cDNA, FLJ95582, highly similar to Homo sapiens breast cancer antiestrogen resistance 1 (BCAR1), mRNA	204	104223	6	0.79	—
Down 78	sp|Q3SXM5-2	—	Isoform 2 of inactive hydroxysteroid dehydrogenase-like protein 1	170	35499	13.5	0.83	Short-chain dehydrogenases of various substrate specificities
Down 79	sp|O43920	NDUFS5	NADH dehydrogenase [ubiquinone] iron-sulfur protein 5	106	16388	11.3	0.74	—
Down 80	tr|H0YG20	MAN1B1	Endoplasmic reticulum mannosyl-oligosaccharide 1,2-alpha-mannosidase (fragment)	155	90816	8.2	0.80	—
Down 81	tr|Q0KKI6	—	Immunoglobulin light chain (fragment)	66	28559	8.2	0.80	—
Down 82	sp|P62244	RPS15A	40S ribosomal protein S15a	1521	18594	66.2	0.82	Ribosomal protein S8
Down 83	tr|B4DL07	—	cDNA FLJ53353, highly similar to ATP-binding cassette subfamily D member 3	398	92669	16.7	0.81	ABC-type uncharacterized transport system, permease, and ATPase components
Down 84	tr|B4DR67	ALG5	Dolichyl-phosphate beta-glucosyltransferase	66	32213	10.9	0.81	Glycosyltransferases involved in cell wall biogenesis
Down 85	tr|Q9BTT5	—	Similar to NADH dehydrogenase (ubiquinone) 1 alpha subcomplex, 9 (39 kD) (fragment)	189	45471	21	0.75	Predicted nucleoside-diphosphate-sugar epimerases
Down 86	tr|Q5U0H8	—	Myelin protein zero-like 1	55	34725	4.8	0.74	—
Down 87	sp|Q5SY16	NOL9	Polynucleotide 5-hydroxyl-kinase NOL9	109	91782	7.4	0.79	Predicted GTPase or GTP-binding protein
Down 88	sp|O15173-2	PGRMC2	Isoform 2 of membrane-associated progesterone receptor component 2	620	30166	26.3	0.75	—
Down 89	sp|Q5VT52-3	RPRD2	Isoform 3 of regulation of nuclear pre-mRNA domain-containing protein 2	295	177879	4.5	0.82	—
Down 90	sp|Q8TC12	RDH11	Retinol dehydrogenase 11	494	41238	14.5	0.76	Dehydrogenases with different specificities (related to short-chain alcohol dehydrogenases)
Down 91	tr|B4DZ55	—	cDNA FLJ52097, weakly similar to Homo sapiens transmembrane and tetratricopeptide repeat containing 1 (TMTC1), mRNA	164	126875	10.1	0.79	FOG: TPR repeat
Down 92	tr|J3KQA9	MTUS2	Microtubule-associated tumor suppressor candidate 2	150	181383	0.6	0.77	—
Down 93	sp|Q96MG7	NDNL2	Melanoma-associated antigen G1	58	41645	7.6	0.72	—
Down 94	tr|H3BQH3	KLHDC4	Kelch domain-containing protein 4 (fragment)	107	47359	10.7	0.83	—
Down 95	tr|J3KN00	NDUFA13	NADH dehydrogenase (ubiquinone) 1 alpha subcomplex, 13	258	28599	23.3	0.81	—
Down 96	sp|Q8NF37	LPCAT1	Lysophosphatidylcholine acyltransferase 1	708	67346	15.7	0.82	1-Acyl-sn-glycerol-3-phosphate acyltransferase
Down 97	sp|Q9Y5P4-2	COL4A3BP	Isoform 2 of collagen type IV alpha-3-binding protein	82	81121	6.7	0.80	—
Down 98	tr|Q5T8U5	SURF4	Surfeit 4	418	22863	39.8	0.81	Predicted membrane protein
Down 99	sp|P26599-2	PTBP1	Isoform 2 of polypyrimidine tract-binding protein 1	570	69515	16.2	0.82	—
Down 100	sp|Q8NC56	LEMD2	LEM domain-containing protein 2	137	63423	7.4	0.76	—
Down 101	tr|Q2Q9H2	G6PD	Glucose-6-phosphate 1-dehydrogenase (fragment)	2165	64315	58.3	0.80	Glucose-6-phosphate 1-dehydrogenase
Down 102	sp|P21796	VDAC1	Voltage-dependent anion-selective channel protein 1	2340	38777	62.9	0.80	—
Down 103	tr|J3KNH7	SENP3	Sentrin-specific protease 3	88	73986	7.7	0.78	Protease, Ulp1 family
Down 104	sp|A6NHL2-2	TUBAL3	Isoform 2 of tubulin alpha chain-like 3	768	51287	11.8	0.79	Tubulin
Down 105	tr|B4DR71	—	cDNA FLJ57078, highly similar to Homo sapiens opioid receptor, sigma 1 (OPRS1), transcript variant 1, mRNA	63	18151	8.4	0.83	—
Down 106	sp|Q5JRA6-2	MIA3	Isoform 2 of melanoma inhibitory activity protein 3	415	249369	7.8	0.80	—
Down 107	tr|J9ZVQ3	APOE	Apolipoprotein E (fragment)	171	30543	12.2	0.79	—
Down 108	tr|G5E9V5	MRPS22	28S ribosomal protein S22, mitochondrial	224	49264	17.3	0.77	—
Down 109	tr|B7Z7X8	ATL2	Atlastin-2	112	76668	10.8	0.82	—
Down 110	sp|P54709	ATP1B3	Sodium/potassium-transporting ATPase subunit beta-3	243	39135	17.9	0.83	—
Down 111	tr|Q6IBK3	SCAMP2	SCAMP2 protein	258	39155	9.7	0.81	—
Down 112	tr|A4LAA3	ATRX	Alpha thalassemia/mental retardation syndrome X-linked	129	374604	2.5	0.81	Superfamily II DNA/RNA helicases, SNF2 family
Down 113	sp|Q9UK59	DBR1	Lariat debranching enzyme	203	72182	14.5	0.80	—
Down 114	tr|B4DI61	—	cDNA FLJ58182, highly similar to protein CYR61	68	50414	6.4	0.70	—
Down 115	tr|H3BNF1	CLN6	Ceroid-lipofuscinosis neuronal protein 6	300	12918	20	0.80	—
Down 116	tr|E7ERK9	EIF2B4	Translation initiation factor eIF-2B subunit delta	170	71199	8.8	0.79	Translation initiation factor 2B subunit, eIF-2B alpha/beta/delta family
Down 117	tr|H0Y8C3	MTCH1	Mitochondrial carrier homolog 1 (fragment)	97	50964	12.9	0.81	—
Down 118	tr|B2RMV2	CYTSA	CYTSA protein	52	149539	2.5	0.79	Ca^2+^-binding actin-bundling protein fimbrin/plastin (EF-hand superfamily)
Down 119	tr|I3L1P8	SLC25A11	Mitochondrial 2-oxoglutarate/malate carrier protein (fragment)	470	37200	35.5	0.83	—
Down 120	sp|Q8NBU5-2	ATAD1	Isoform 2 of ATPase family AAA domain-containing protein 1	124	40468	11.1	0.72	ATPases of the AAA+ class
Down 121	sp|Q9Y3E7	CHMP3	Charged multivesicular body protein 3	102	32415	14.4	0.83	Conserved protein implicated in secretion
Down 122	sp|P02763	ORM1	Alpha-1-acid glycoprotein 1	262	28288	20.4	0.80	—
Down 123	tr|Q53F51	—	FGF intracellular binding protein isoform b variant (fragment)	165	48798	12	0.83	—
Down 124	sp|Q3ZAQ7	VMA21	Vacuolar ATPase assembly integral membrane protein VMA21	241	12868	24.8	0.81	—
Down 125	tr|B2R6X8	—	cDNA, FLJ93169, highly similar to Homo sapiens GPAA1P anchor attachment protein 1 homolog (yeast) (GPAA1), mRNA	106	72151	7.6	0.80	—
Down 126	sp|Q9P0S9	TMEM14C	Transmembrane protein 14C	45	12774	8.9	0.70	—
Down 127	sp|P08779	KRT16	Keratin, type I cytoskeletal 16	630	57054	23.9	0.62	—
Down 128	sp|Q86UT6-2	NLRX1	Isoform 2 of NLR family member X1	75	110309	4.1	0.71	—
Down 129	tr|Q59E99	—	Thrombospondin 1 variant (fragment)	153	155789	3.4	0.68	—
Down 130	sp|Q8WXH0-2	SYNE2	Isoform 2 of nesprin-2	149	986758	1.1	0.82	Ca^2+^-binding actin-bundling protein fimbrin/plastin (EF-hand superfamily)
Down 131	sp|P78310-2	CXADR	Isoform 2 of coxsackievirus and adenovirus receptor	47	47491	3.8	0.74	—
Down 132	tr|B2R995	—	Malic enzyme	98	77738	5.8	0.83	Malic enzyme
Down 133	tr|Q5QP56	BCL2L1	Bcl-2-like protein 1 (fragment)	98	21810	23.2	0.82	—
Down 134	tr|H0YK72	SEC11A	SEC11-like 1 (*S. cerevisiae*), isoform CRA_a	247	22018	16.5	0.81	Signal peptidase I
Down 135	tr|B4DDH8	—	cDNA FLJ55184, highly similar to Homo sapiens leukocyte receptor cluster (LRC) member 4 (LENG4), mRNA	137	54865	8.8	0.79	Predicted membrane protein
Down 136	sp|Q9UJS0-2	SLC25A13	Isoform 2 of calcium-binding mitochondrial carrier protein Aralar2	719	86824	17.5	0.82	—
Down 137	tr|A8KAK5	—	cDNA FLJ77399, highly similar to Homo sapiens cofactor required for Sp1 transcriptional activation, subunit 2, 150 kDa (CRSP2), mRNA	85	182987	3.4	0.82	—
Down 138	tr|H0YEF3	RNASEH2C	Ribonuclease H2 subunit C (fragment)	76	18856	25.3	0.77	—
Down 139	tr|Q5QNZ2	ATP5F1	ATP synthase F(0) complex subunit B1, mitochondrial	406	27794	47.7	0.82	—
Down 140	sp|Q6UW68	TMEM205	Transmembrane protein 205	165	23294	15.9	0.82	—
Down 141	tr|B3KPJ4	PHC2	Polyhomeotic-like protein 2	193	59764	9.3	0.79	—
Down 142	tr|H0Y4D4	ACAA1	3-Ketoacyl-CoA thiolase, peroxisomal (fragment)	131	30218	12.7	0.78	Acetyl-CoA acetyltransferase
Down 143	tr|Q4G0F4	POLRMT	DNA-directed RNA polymerase	167	159664	4.6	0.81	Mitochondrial DNA-directed RNA polymerase
Down 144	tr|Q6FGZ3	EPHX1	EPHX1 protein (fragment)	519	62281	14.9	0.77	Predicted hydrolases or acyltransferases (alpha/beta hydrolase superfamily)
Down 145	tr|B4DVN1	—	cDNA FLJ52214, highly similar to DnaJ homolog subfamily B member 6	90	37740	8.6	0.70	DnaJ-class molecular chaperone with C-terminal Zn finger domain
Down 146	sp|Q92667-2	AKAP1	A-kinase anchor protein 1, mitochondrial	66	111940	4.9		
Down 147	sp|O00483	NDUFA4	NADH dehydrogenase [ubiquinone] 1 alpha subcomplex subunit 4	165	11855	46.9	0.83	—
Down 148	sp|Q9NTJ5	SACM1L	Phosphatidylinositide phosphatase SAC1	179	77476	18.2	0.83	Phosphoinositide polyphosphatase (Sac family)
Down 149	tr|B3KVC5	—	cDNA FLJ16380 fis, clone TLIVE2002882, weakly similar to imidazolonepropionase (EC 3.5.2.7)	41	53582	3.3	0.83	Imidazolonepropionase and related amidohydrolases
Down 150	tr|B7ZLI5	FAM98C	Family with sequence similarity 98, member C	72	41696	9.5	0.68	—
Down 151	tr|B7Z6F5	YIPF1	Protein YIPF1	64	40866	2.7	0.61	—
Down 152	sp|Q6NVY1-2	HIBCH	Isoform 2 of 3-hydroxyisobutyryl-CoA hydrolase, mitochondrial	101	46543	19.2	0.82	Enoyl-CoA hydratase/carnitine racemase
Down 153	tr|U3KQJ1	POLDIP2	Polymerase delta-interacting protein 2	282	46395	26.4	0.76	Uncharacterized protein affecting Mg^2+^/Co^2+^ transport
Down 154	tr|D6RGZ2	THOC3	THO complex subunit 3	172	12690	36.2	0.75	—
Down 155	tr|A0S0T0	ATP6	ATP synthase subunit a	128	26896	4.4	0.78	F0F1-type ATP synthase, subunit a
Down 156	tr|G3V2U7	ACYP1	Acylphosphatase	85	17520	14.7	0.80	acylphosphatases
Down 157	sp|Q9ULG6-2	CCPG1	Isoform 2 of cell cycle progression protein 1	79	93313	4.1	0.81	—
Down 158	tr|H7BXZ6	RHOT1	Mitochondrial Rho GTPase	142	81600	5.9	0.77	GTPase SAR1 and related small G proteins
Down 159	sp|Q14151	SAFB2	Scaffold attachment factor B2	461	129824	13	0.83	—
Down 160	sp|Q96LD4	TRIM47	Tripartite motif-containing protein 47	138	75838	7.8	0.81	—
Down 161	tr|A8K2K2	—	cDNA FLJ76494, highly similar to Homo sapiens GTPBP2 GTP-binding like protein 2	137	64767	11.7	0.83	GTPase

## Abbreviations

iTRAQ: Isobaric tag for relative and absolute quantification
KEGG: Kyoto Encyclopedia of Genes and Genomes
COG: Cluster of orthologous groups of proteins
Go: Gene Ontology
FBS: Fetal bovine serum
SCX: Strong cation exchange
HCD: High-energy collision dissociation
AGC: Automatic gain control
NR: Nonredundant protein database
SDS-PAGE: Sodium dodecyl sulfate polyacrylamide gel electrophoresis
ECL: Enhanced chemiluminescence
BP: Biological process
CC: Cellular component
MF: Molecular function
RPs: Ribosomal proteins
MTP: Mitochondrial transmembrane potential
Vps34: Vacuolar protein sorting-34
UVRAG: UV-resistance-associated gene
AMBRA-1: Activating molecule in Beclin-1-regulated autophagy.
